# Mechanism of Self-Assembly of the Gonadropin Releasing Hormone Antagonist Teverelix into Amyloid Fibrils

**DOI:** 10.1021/acs.molpharmaceut.5c00578

**Published:** 2025-12-18

**Authors:** Xinyang Li, Louise C. Serpell, Jens T. Bukrinski, Francois Boutignon, Carol M. MacLean, Sophie E. Jackson

**Affiliations:** † 150385 Yusuf Hamied Department of Chemistry, Lensfield Road, Cambridge CB2 1EW, U.K.; ‡ Sussex Neuroscience, School of Life Sciences, 1948 University of Sussex, Brighton BN1 9QG, U.K.; § CMCAssist ApS, Ole Maalo̷es Vej 3, Copenhagen N 2200, Denmark; ∥ Boutignon & Partners, 2 rue Michel Renaud, Biopole Clermont-Limagne, Saint Beauzire 63360, France; ⊥ Antev Ltd, Ibex House, Baker Street, Weybridge, Surrey KT13 8AH, U.K.

**Keywords:** slow-release depot, amyloid fibrils, nucleation−polymerization mechanism, GnRH antagonist, prostate cancer, therapeutic peptide

## Abstract

Teverelix is a short non-natural peptide, which is a gonadotropin releasing hormone antagonist and used as a treatment for prostate cancer. Teverelix is formulated as a trifluoroacetic acid salt, which, at the high concentrations used for parenteral injection, forms a microcrystalline suspension. At low concentrations and immediately after injection, teverelix self-assembles into a fibrillar species thought to be important for the slow-release kinetics and long-acting action of this peptide in vivo. In this paper, we confirmed the amyloid-like identity of teverelix fibrils using X-ray fiber diffraction and transmission electron microscopy. The inter-β-sheet packing distance was found to be larger than that of typical amyloid fibrils and this was attributed to the large non-natural side chains within the peptide. Using data from numerous biophysical experiments, a model of the structure of teverelix within the fibril is proposed. The kinetics of fibril formation were investigated using standard ThT assays, and teverelix found to fibrillate rapidly over a wide range of conditions. The fibrillation rate was shown to depend critically upon pH, peptide, and trifluoroacetic acid concentration. Fibrillation was accompanied by a drop in pH, which we attribute to the fact that the pyridinium side chain must be deprotonated before self-assembly. Based on our results, we propose a nucleation–polymerization mechanism in which dimers of teverelix rapidly self-assemble into amyloid-like fibrils with little change in the secondary structure but burial of some of the aromatic acid side chains. Interestingly, the fibrils can, under certain conditions, align to create a highly ordered array. To the best of our knowledge, this is the first paper studying teverelix in detail from a biophysical perspective, and it is directly relevant to the aggregation of the peptide observed in vivo.

## Graphical abstract

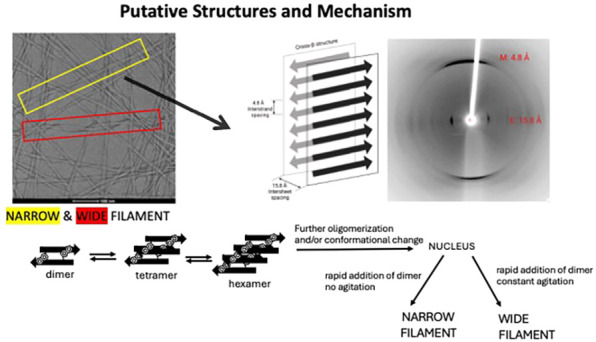

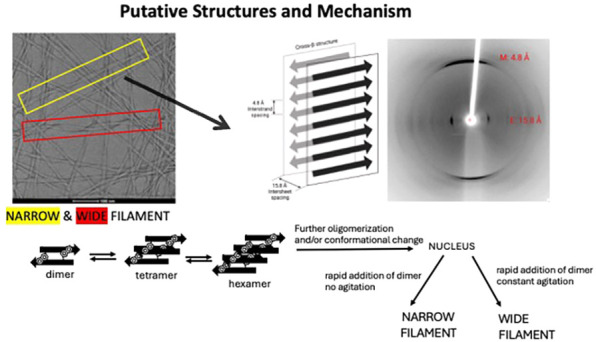

## Introduction

The gonadotropin releasing hormone (GnRH) antagonists are short peptide analogues of GnRH, which bind and block the action of GnRH receptors directly, with a rapid decrease in luteinizing hormone, follicle-stimulating hormone, and testosterone in men and estradiol in women.^1–4^ This is therapeutically valuable for conditions that are controlled by sex hormone secretion, including prostate cancer, benign prostatic hyperplasia, and endometriosis.^5–7^ Teverelix (Tv) is a decapeptide GnRH antagonist that has been developed as an effective treatment for prostate cancer, which is currently under phase III clinical trials.^1,2,4,8^

Teverelix is formulated as a trifluoroacetic acid (TFA) salt, which, at the high concentrations used for injection, forms a microcrystalline suspension. This unusual behavior has only been observed for a few peptides and sometimes only with specific counterions. For example, the acetate salt of teverelix does not form a microcrystalline state.^9^ For other systems like insulin, the microcrystalline suspensions have already been used as a formulation method.^10–12^ This state is essential for the effective formulation and use of teverelix as it enables a solution of the drug at high concentration in a small volume to be injected into a patient.^1,2,4,8^

After subcutaneous injection, teverelix exhibits a biphasic, long-acting release profile that is attributed to two processes. It is known that there is rapid aggregation of teverelix at the site of injection resulting in the formation of a depot, which acts as a slow-release mechanism.^1,2,4,8^ In addition, some of the teverelix is absorbed quickly into the bloodstream, either because it is a state that can be rapidly transported into the circulation or it simply has not yet had the time to aggregate along with the rest of the teverelix. In either case, this leads to the initial rapid release of some of the teverelix, followed by the slow release of more teverelix from the depot.^1,2,4,8^ As other GnRH antagonists have been shown to form amyloid-like fibrils under specific conditions, it is thought, but not known, that this depot may be amyloid in nature.^13–16^

Many peptides, including a significant number of therapeutic peptides, are known to aggregate when formulated at high concentrations, this being one of the most common and troubling processes encountered in almost all phases of biological drug development.^17–19^ Aggregates can be amorphous or highly structured, for example, amyloid fibrils.^20–22^ In some cases, it has been shown that the reduction of physical stability of peptide/protein-based therapeutics not only leads to loss of activity, but also causes other, potentially severe, problems such as toxicity and immunogenicity.^23–25^ However, in other cases, aggregation into amyloid fibrils in particular may have certain advantages. For example, the use of amyloids in a pharmaceutical application for the formulation of long-acting drugs (through the formation of an amyloid depot) has been explored.^16^ In the case of teverelix, understanding the formation of an amyloid-like slow-release depot, which is formed in vivo, is essential for optimizing its therapeutic use and efficacy. Despite this, there have been no detailed studies on the self-assembly of teverelix or the factors that govern which physical state dominates.^26^ The single published study simply determined the critical aggregation concentration of teverelix under one condition.^26^ Until now, the focus has been on its pharmacological behavior.^27–29^

Here, we report a comprehensive study of the self-assembly of teverelix into a fibrillar state at concentrations between 0.05 and 10 mg/mL using multiple different biophysical approaches. N.B. We do not study the formation of the microcrystalline state here, which requires much higher peptide concentrations. We show that the peptide does form amyloid fibrils under a wide range of conditions, despite its unusual sequence, which contains both L- and D-amino acids as well as unnatural and, in some cases, large side chains. Kinetic studies were employed to understand the rate and mechanism by which teverelix forms amyloid fibrils, and transmission electron microscopy experiments provided detailed structural information about the morphology of fibrils and higher-order structures formed.

In this study, a range of spectroscopic methods were employed to probe the aggregation of teverelix, including thioflavin T (ThT) assays. ThT is capable of binding to the cross-β sheet structure of amyloid fibrils, and, in this case, an increase in its fluorescence intensity at around 480 nm is observed; therefore, it has been widely used as a method to monitor fibrillation processes.^30^ However, it should be noted that there are a considerable number of cases where an increase in ThT fluorescence is not due to the formation of fibrils, so additional methods to verify fibril formation are essential.^30^ X-ray fiber diffraction (XRD) was used to determine whether the Tv fibrils are amyloid or not,^31,32^ and the fibrils were further characterized using transmission electron microscopy (TEM) and various spectroscopic techniques. In addition, the starting state (freshly prepared solutions of teverelix) was investigated using different experimental methods and shown to be dimers with similar secondary structure to the fibrils, explaining the rapid fibril formation observed under some conditions.

Our studies do not start with the normal design of buffer experiment as teverelix has already undergone several clinical trials. Instead, and somewhat unusually, we start by studying the self-assembly of teverelix without buffer, as this is how it is currently formulated in clinical trials. This has the advantage of (i) better mimicking the formulation conditions and, importantly, (ii) enabling us to detect whether there are any protonation/deprotonation processes associated with fibril formation. Subsequently, buffered conditions were employed to gain further understanding of how Tv concentration, pH, and TFA and NaCl concentrations affected the rate of self-assembly.

## Material

Teverelix (Tv), Ac-D-Nal­(2)-d-Phe­(4Cl)-D-Pal­(3)-Ser-Tyr-D-Hcit-Leu-Lys­(iPr)-Pro-d-Ala-NH_2_ in the form of a TFA salt (white powder), was supplied by Antev (Antev Ltd., UK) with a purity of 93.2% and a molecular weight of 1460 kDa. Tv was stored in a −20 °C freezer and used without further purification.

## Methods

### Sample Preparation and Incubation

Tv powder was dissolved in the appropriate buffer in a 1.5 mL Eppendorf tube (Eppendorf International, Germany). To aid the dissolution of Tv, the samples were transferred into a Thermomixer compact (Eppendorf International, Germany) with 300 rpm agitation at 37 °C for 30 s. The concentration of the Tv solution was determined spectroscopically on a Cary 60 UV–vis spectrophotometer (Agilent Technologies, USA) by using the Beer–Lambert Law and a calculated extinction coefficient of 5426 M^–1^ cm^–1^ at 280 nm. The Tv samples were sealed with paraffin film (Fisher Scientific, USA) and covered with aluminum foil to prevent solvent evaporation and exposure to sunlight. Incubation was performed at room temperature without further agitation, unless in the ThT assays or as stated otherwise.

### Intrinsic Fluorescence

The intrinsic fluorescence measurements were performed on a Cary Eclipse fluorescence spectrophotometer (Agilent Technologies, USA). Spectra were acquired from 300 to 400 nm using an excitation wavelength of 280 nm and a wavelength step of 1.0 nm. Both excitation and emission bandpasses were kept at 10 nm with an appropriate voltage on the photomultiplier tube ranging from 550 to 750 V. All samples were measured in a 120 μL quartz cuvette (Hellma Analytics, Germany) at 25 °C.

### Circular Dichroism (CD)

The circular dichroism measurements were performed on a Chirascan CD spectrophotometer (Applied Photophysics, UK). All samples were measured with a 1 nm wavelength step and 1 nm spectral bandwidth at 25 °C. The far-UV CD spectra were acquired from 190 to 250 nm with samples in a 0.1 mm (or 0.01 mm) path length cuvette, while near-UV CD spectra were acquired from 250 to 350 nm with samples in a 0.2 mm path length cuvette. The result for each measurement was obtained by averaging three scans, followed by the subtraction of the buffer background.

### Transmission Electron Microscopy (TEM)

2.5 μL of Tv samples with appropriate concentrations and conditions was spotted onto a glow-discharged, carbon-coated copper grid (Agar Scientific, UK) for 30 s. The glow discharge was performed on a Quorum Technologies (UK) GloQube system prior to the sample application. The excess sample solution was removed by blotting the edge of the grid with a filter paper. The sample was further stained by loading 2.5 μL of 2% (w/w) aqueous uranyl acetate solution onto the grid for 1 min followed by the removal of excess staining fluid with a filter paper. 2.5 μL of water was then used to wash the grid to remove the salts presented, and the grid was dried in air. TEM analysis was performed using a Thermo Scientific (USA) Talos F200X G2 transmission electron microscope with an acceleration voltage of 200 kV.

### Thioflavin T Assays (ThT Assays)

Thioflavin T assays were performed on a microplate reader FLUOstar Omega (BMG Labtech, Germany). Samples were transferred into a 96-well half area plate (Corning 3881, USA) and sealed with tape (Costar Thermowell) to prevent the evaporation of samples. Samples were incubated in the plate reader at 37 °C with a final concentration of ThT of 50 μM. ThT fluorescence was measured using an excitation filter at 440 nm and an emission filter at 480 nm. Bottom reading of the plate was performed every 30 min with 5 min shaking (orbital shaker at 600 rpm) prior to each measurement. Fluorescence was measured at a gain of 500 with 8 flashes per well.

### Sigmoidal Fitting of ThT Assays

The results from ThT binding assays were fitted with the following equation:

$$y=y_{0}+\frac{A}{1+\exp(-k(t-t_{1/2}))}+b\cdot t$$ 1

where *y*_0_ is the initial fluorescence, *A* is the amplitude of the transition, *t*_1/2_ is the half-time (the time at which the ThT fluorescence reaches half of the final plateau value), *k* is the apparent growth rate, and *b* is the slope of the final baseline. The lag time was calculated using the parameters obtained from the best fit of the data to eq 1, using the following equation:

$$t_{\mathrm{lag}}=t_{1/2}-\frac{2}{k}$$ 2

### Thioflavin T Fluorescence

ThT fluorescence was measured on a Cary Eclipse fluorescence spectrophotometer (Agilent Technologies, USA). Spectra were acquired using an excitation wavelength of 448 nm, and emission was recorded from 460 to 600 nm with a wavelength step of 1 nm. The final concentration of ThT was kept at 50 μM, which was the same as that used in the ThT assays. Both excitation and emission bandpasses were kept at 10 nm with an appropriate voltage on the photomultiplier tube ranging from 550 to 750 V.

### Size-Exclusion Chromatography

Analytical size-exclusion chromatography (SEC) was performed using an AKTA/FPLC system (GE Healthcare, USA) with a Superose 12 10/300 column (GE Healthcare, USA). A 200 μL injection loop was used for sample loading. All samples were filtered through 0.22 μm filters before being loaded onto the column. All samples were eluted with a flow rate of 0.75 mL min^–1^ at room temperature and an upper pressure limit of 3 MPa. The elution process for each run was monitored by using a UV absorbance detector at 280 nm through a 0.5 cm flow cell.

### X-ray Fiber Diffraction

Tv samples (5 mg/mL) were prepared in 25 mM citrate buffer (pH 3.0) and incubated at room temperature for 1 week without shaking. For comparison, samples were incubated at room temperature without shaking and in an incubator at 37 °C with shaking for a week. Tv fibrils were prepared by hanging 10 μL droplets of Tv between two wax-tipped capillaries, followed by overnight drying (also known as the alignment procedure). The dried Tv fibrils (Figure S1) were transferred into a diffractometer (Malvern Panalytical, UK) with care to avoid breakage of the fibril bundle samples. The recorded X-ray diffraction patterns were converted to the JPEG format and subsequently analyzed by X-ray fiber diffraction analysis program CLEARER^33^ to extract the values of the equatorial and meridional reflections.

## Results and Discussion

Teverelix (Tv) is a synthetic peptide GnRH antagonist, featuring an unusual primary structure. It has several large, unnatural side chains (Nal, Cpa, Pal, hCit, and Lys­(iPr)) as well as being comprised of both L- and D-amino acids (Figure 1A). Here, we show the results of studies on the self-assembly of Tv over a range of peptide concentrations from 0.05 to 10 mg/mL and from pH 2.7 to 5.0. In some cases, the peptide solutions contained no buffer (to mimic the conditions currently used in the drug formulation and to probe changes in pH during self-assembly). In other cases, an appropriate buffer was used to maintain the pH of the solution throughout self-assembly.

**Figure 1:**
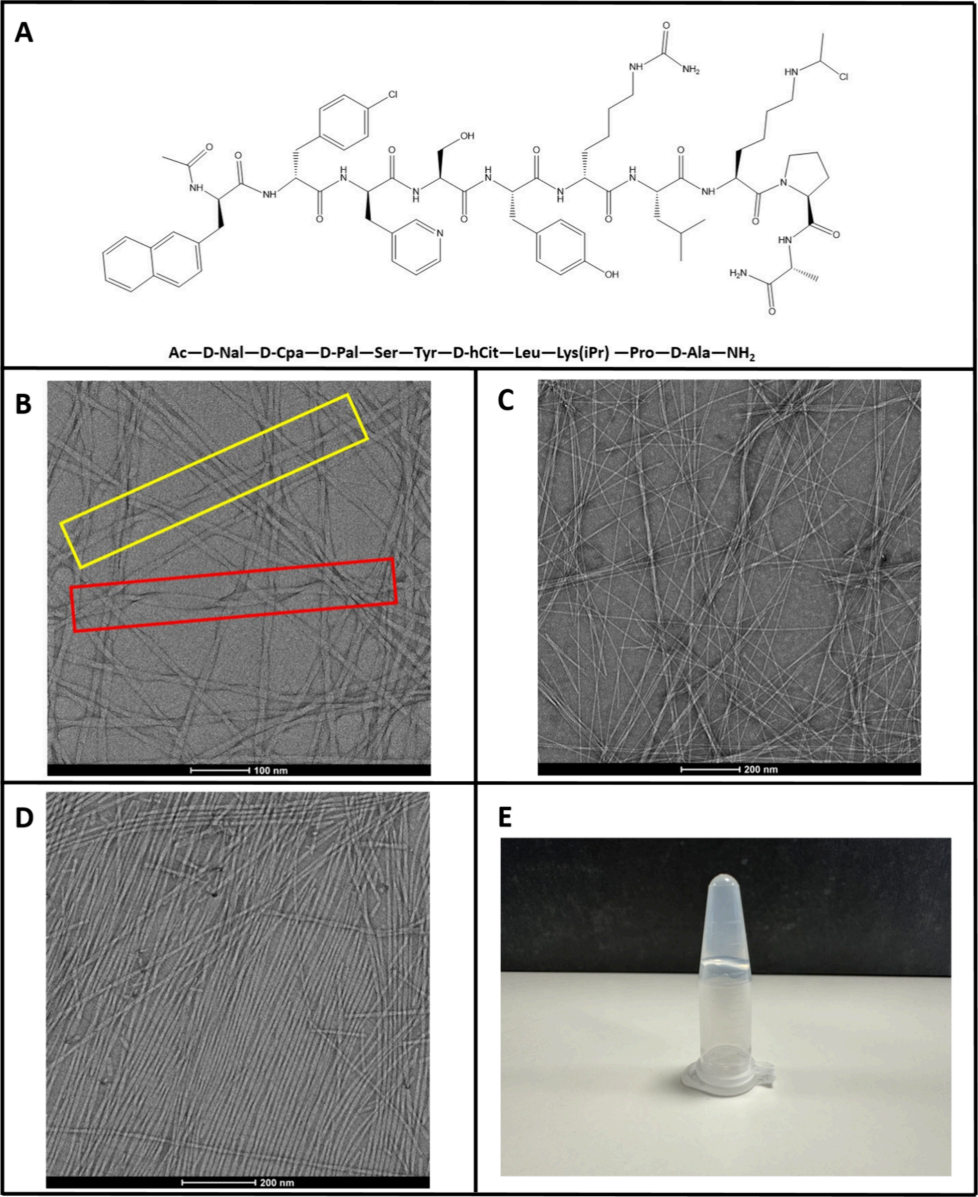
Chemical structure of teverelix and images of the fibrillar states it adopts. (A) Chemical structure of teverelix. (B) TEM image of the fibrils formed by a 2 mg/mL solution of Tv in dd H_2_O incubated for 7 days at 37 °C. A typical narrow filament is shown in the yellow rectangle (diameter 10 nm), and a wide filament (diameter 20 nm) assembled from two narrow filaments is shown in the red rectangle. (C) TEM image of a network of Tv fibrils in a gel-like sample. One mg/mL Tv in 25 mM citrate incubated for 14 days at 37 °C was used (pH 3.0). (D) Alignment of Tv fibrils as observed in a 3 mg/mL solution of Tv in ddH_2_O incubated for 14 days at 37 °C (pH 2.73). (E) Inverted microcentrifuge tube showing the gel-like sample of Tv formed by 10 mg/mL Tv in 25 mM citrate incubated for 14 days at 37 °C (pH 3.0).

### Teverelix Forms Fibrils over a Range of Conditions as Shown by Transmission Electron Microscopy

In our initial experiments, solutions of Tv were found to form fibrils over a range of different peptide concentrations from 1 to 3 mg/mL in both unbuffered and buffered solutions from pH 2.73 to 3.5 after incubation for 1–2 weeks. Figure 1B–D shows transmission electron microscopy (TEM) images of the fibrils formed. In these cases, the samples were incubated at room temperature under quiescent conditions; the process did not, therefore, require elevated temperatures, pressures, or agitation. For solutions of 0.2 mg/mL Tv at pH 4.1 and 1.0 mg/mL Tv at pH 3, fibrils were observed directly in freshly prepared samples after approximately only 30 min of incubation at room temperature, Figure S1A,B, establishing that fibril formation can be rapid.

Two types of fibrils with different widths were observed by TEM (shown in the yellow and red boxes in Figure 1B). Under conditions used for most of the X-ray fiber diffraction and TEM microscopy experiments, samples were incubated at room temperature under quiescent conditions. Narrow filaments of Tv were the major species observed (width 10 nm), while occasionally, larger wide Tv filaments were also observed (width 20 nm), which appeared to be twisted pairs of narrow filaments. Both were twisted into a helix with a repeat approximately 100 nm. Thus, the dimensions of the fibrils of teverelix are consistent with those observed for other amyloid fibrils.^34,35^ Interestingly, wide filaments were the dominant species when samples of Tv were incubated at 37 °C with constant agitation (Figure S1C,D). However, under the conditions used in all of the experiments described here, including the kinetic experiments in which there was periodic but not constant agitation, narrow filaments were the dominant species. In the rest of the paper, the narrow filaments will be referred to as fibrils. All of the fibrils observed within these studies had the same overall structure and morphology in terms of widths and twist.

### Teverelix Fibrils Can Form Different Higher-Order Structures: Networks and Aligned States

At sufficiently high Tv concentrations and over time, the physical state of Tv samples changed from a nonviscous, solution-like state to a viscous gel-like state as self-assembly occurred (Figure 1E). In the gel-like state (formed at higher Tv concentrations with long incubation times), TEM was used to image the sample, which showed that a high concentration of fibrils formed a random network in which fibrils cross over each other (Figure 1C), like many other fibril-forming peptides.^36^ Under different conditions (moderate Tv concentration with incubation times of approximately 2 weeks), the fibrils formed by Tv were found to strongly align forming a much less heterogeneous state (Figure 1D). Although there was a pipetting step in the preparation of the TEM grid, the alignment of Tv fibrils did not appear to need any additional external force, e.g., flow of the solvent as is the case for a number of other peptides.^37–39^ It should be noted that the alignment was not observed in every sample, even though every sample prepared for TEM had a pipetting step, suggesting that this result is not an artifact of the pipetting step but that Tv fibrils align in solution only under a specific set of conditions.

The TEM results show that Tv self-assembles into fibrils over a wide range of conditions and that the fibrils formed have dimensions like those observed in amyloid fibrils.^34^ They also establish that fibrils of Tv can adopt different higher-order structures, forming either a network of criss-crossed filaments or fibrils that are highly aligned. Despite the power of TEM to image the fibrils of Tv, these results do not prove that the fibrillar state of Tv is an amyloid.

### Teverelix Forms Amyloid-like Fibrils as Shown by X-ray Fiber Diffraction

Amyloid fibrils are well-known to display a cross-β diffraction pattern by X-ray fiber diffraction.^31,32,40^ A sample of Tv fibrils was aligned to form a bundle of fibrils, and an X-ray diffraction pattern was collected (Figure 2A). The well-oriented diffraction pattern shows a strong 4.8 Å meridional reflection arising from the hydrogen bonding distance between β-strands in a β-sheet. Several weak reflections were observed on the equator, as well as a strong, sharp 15.3 Å equatorial reflection, which was interpreted to arise from the spacing between the β-sheets. From these values, a schematic showing arrangement of the peptide and the wide β-sheet spacing was constructed and is shown in Figure 2B.

**Figure 2:**
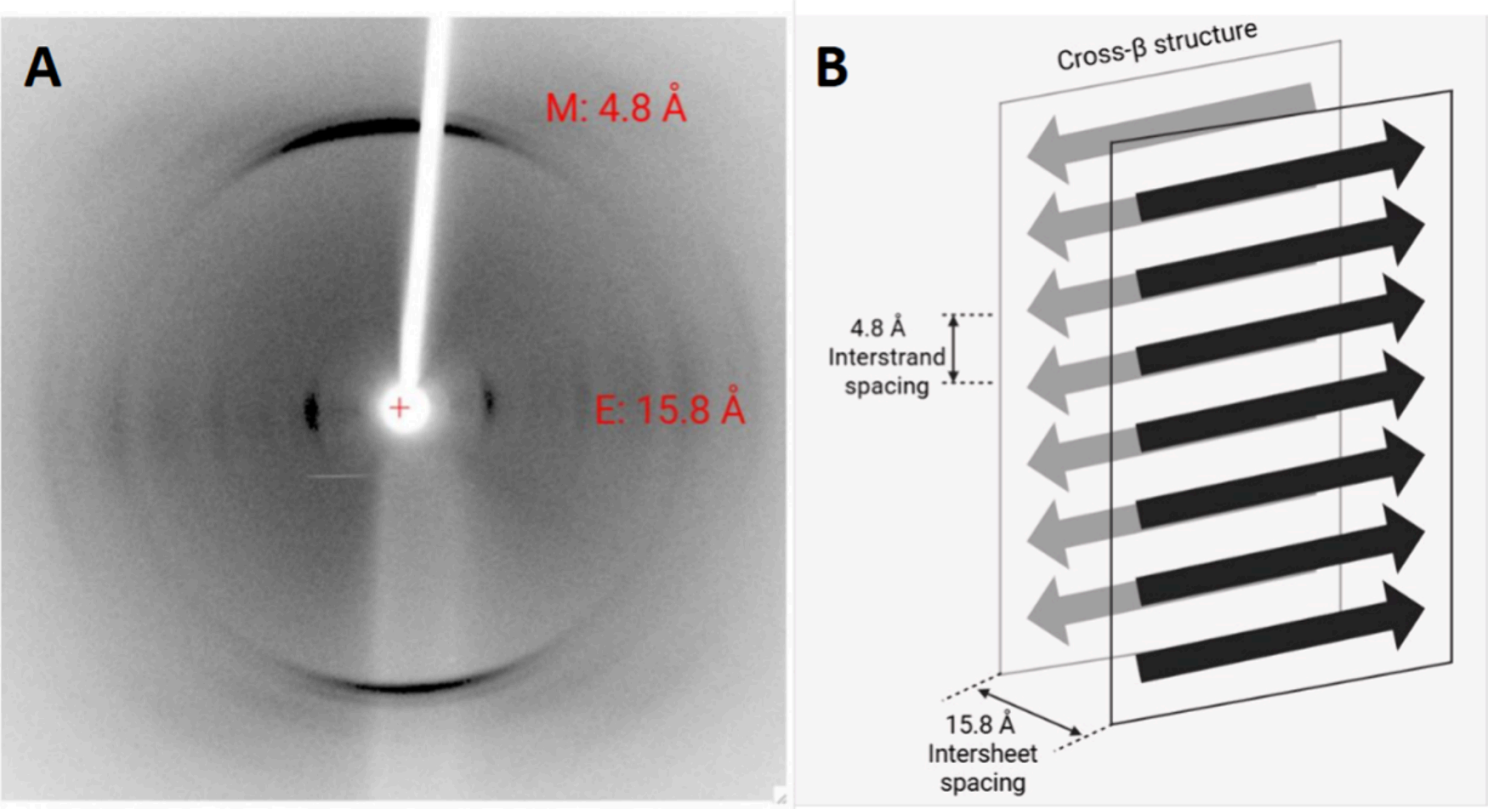
X-ray fiber diffraction pattern and model of β-sheet spacing. (A) X-ray fiber diffraction pattern of 5 mg/mL solution of Tv in 25 mM citrate incubated for 7 days at 37 °C without agitation (pH 3.0). The meridional (M) and equatorial (E) reflections extracted are shown in red, which were 4.8 and 15.8 Å, respectively. (B) Model of the β-sheet spacing of the typical cross-β structure for amyloid. The interstrand and intersheet spacings are indicated (4.8 and 15.8 Å) based on the values obtained in A.

The diffraction pattern is consistent with cross-β conformation, although the equatorial reflection is larger than is typical (∼12.0 Å).^40^ The sharp reflections in both the meridional and equatorial axes suggest a highly ordered structure for the peptide within the fibrils. Since there are several bulky side chains in the Tv structure (Figure 1A), which will impact the stacking of the β-sheets within the fibril, the difference in equatorial reflection is reasonable, and we conclude that Tv forms amyloid-like fibrils.

### pH Decreases during the Formation of Amyloid Fibrils

Initial experiments on the self-assembly of Tv focused on the behavior of unbuffered solutions of the peptide, as this more closely mimics the current formulation condition of the peptide. In these cases, the acidic counterion TFA content largely determines the pH of the solution, and therefore, it is expected that the pH varies with the peptide concentration. Using unbuffered solutions enabled experiments to determine if there is any change in the protonation state of the peptide during self-assembly. The pH of the Tv samples was measured both prior to, and after, aggregation, both in Tv samples made up in ddH_2_O as well as those dissolved in a buffer to control the pH (to ensure that buffer concentrations were sufficient to maintain the pH throughout the self-assembly process when buffers were used). The results are shown in Figure 3A and Table S1.

**Figure 3:**
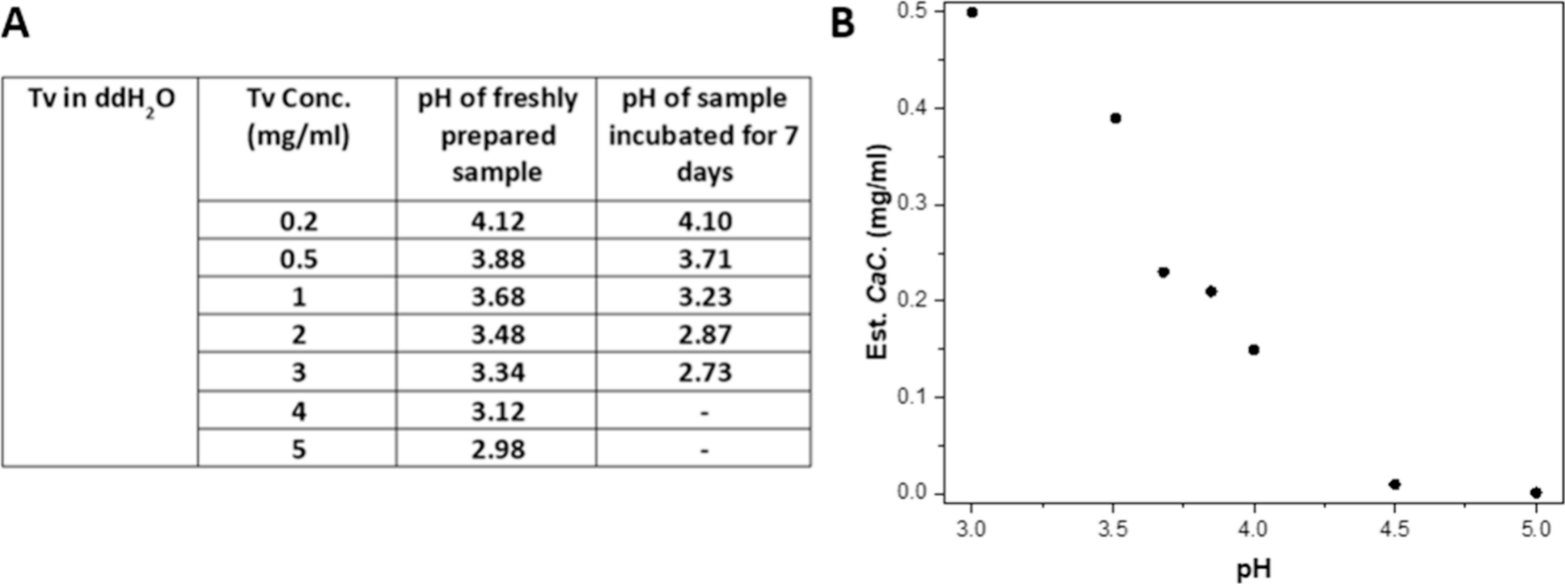
pH decrease of Tv upon incubation and estimated critical aggregation (cac) from pH 3.0 to 5.0. (A) pH values of freshly prepared and seven-day incubated Tv samples from 0.2 to 5 mg/mL in ddH_2_O at room temperature. Due to the high viscosity of gel-like samples formed, the pH values of 4 and 5 mg/mL Tv in ddH_2_O incubated for 7 days were not measured. (B) Estimated critical aggregation concentration of freshly prepared Tv in 25 mM citrate at 25 °C (pH from 3.0 to 5.0). A series of Tv (0.05–1 mg/mL) was freshly prepared in 25 mM citrate and measured in order to obtain the corresponding cac values (pH 3.0–5.0). For more details, see Figures S2–S4.

For the nonbuffered samples, the initial pH recorded immediately after preparation of the sample was found to vary with the concentration of the peptide as expected as the TFA counterion is acidic. In addition, the pH was observed to decrease significantly after self-assembly, as shown in Figure 3A. These results indicate that a deprotonation step is associated with the formation of the Tv amyloid fibrils. Calculations establish that a single deprotonation event occurs (see the footnote to Table S1). Considering the p*K*_a_ of all possible protonation sites in Tv, we think that the pyridine side chain, which has a p*K*_a_ of 5.22, is the most likely one to be involved; however, we cannot completely rule out the possibility that it is another side chain with a perturbed p*K*_a_ value. At the low pH values used in this study, the pyridine side chain will be in a protonated form (pyridinium ion) and will carry a positive charge at the start of the reaction. The results show that deprotonation (and elimination of the positive charge) of the pyridinium group is essential for the self-assembly process to occur. Thus, it is highly likely that the pyridine side chain is buried in the fibrillar state, potentially acting as a H-bond acceptor. These results suggest that the self-assembly of Tv is highly likely to be pH-dependent.

Deprotonation/protonation events on fibril formation have been observed before in studies on other peptides including glucagon.^44^ In this case, depending on whether the peptide is in acidic or alkaline conditions, it either protonates or deprotonates to have a net charge of zero in the fibrillar state. Thus, this study established that the p*K*_a_ of side chains can be dramatically different in the fibrillar state compared to in solution.^44^

### Critical Aggregation Concentration for Tv Self-Assembly Is pH-Dependent

To establish whether the self-assembly of Tv is pH-dependent, the critical aggregation concentration (cac) of Tv was estimated by using ThT fluorescence. Although the ThT dye can bind to a number of non-amyloid-like states, in cases where amyloid formation has been shown by other methods, e.g., X-ray fiber diffraction, ThT is a more reliable measure of the extent of amyloid fibril formation and has been used by many groups as a measure of the amount of amyloid fibrils in solution.^30^ ThT fluorescence of a series of Tv samples (0.01–1 mg/mL) in buffered solutions over a range of pH values from 3.0 to 5.0 were measured (Figures S2–S4). ThT dye was added to each sample prior to the fluorescence measurement, and the cac was estimated from the data. It should be noted that the cac values are estimates, as the system is not in equilibrium. They can be considered the cac values after 1 h of incubation at room temperature. Over longer periods of time, the starting state will further convert into fibrils and the apparent cac will therefore decrease over time. Despite this, the values still give a clear indication of how fibril formation depends on pH. The data show that the cac values decrease as the pH increases from 3 to 4.5, reaching a plateau between pH 4.5 and 5.0 (Figure 3B), suggesting that the fibrillar form is more stable at higher pH values. These results are consistent with the decrease in pH observed on fibril formation as discussed above, and the pyridinium side chain being deprotonated in the fibrillar state. Given that the stability of the fibrils depends critically on pH, it is highly likely that the kinetics of fibril formation will also be pH-dependent.

### Kinetics of Tv Amyloid Fibril Formation

The kinetics of fibril formation for Tv were investigated using ThT assays over a wide range of conditions and pH values using buffered solutions. It is well-known in the literature that an increase in ThT fluorescence can occur even in the absence of amyloid-like fibrillar species. Therefore, for many of our kinetic runs, TEM imaging was employed at the end of the ThT assay to confirm the presence of fibrillar species (Figure S5), thus establishing that ThT is a reliable indicator of fibril formation.

At low Tv concentrations and/or low pH (pH 3.0–4.0), sigmoidal growth curves showing a lag and growth phase followed by a plateau were observed (Figure 4A,B). Where possible, the ThT curves were fit to extract the key kinetic parameters—the lag time (*t*_lag_), *t*_1/2,_ the time at which the ThT signal is 50% of its final value, and *k*, the apparent growth rate, which corresponds to the steepest part of the growth phase. The effects of peptide concentration, pH, TFA concentration, and mannitol (which is present in the formulation of Tv) were all investigated to obtain information about the factors affecting the rate of fibril formation as well as mechanistic insight.

**Figure 4:**
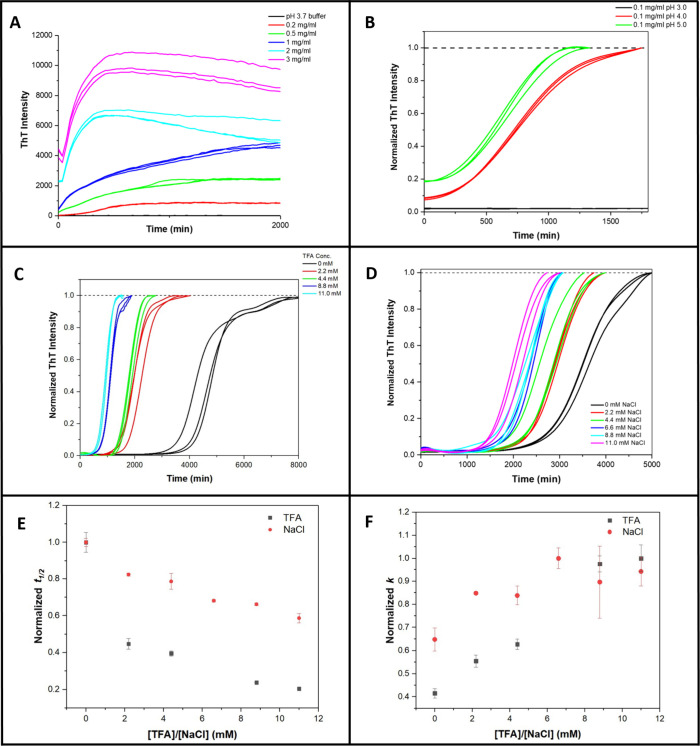
Kinetic data from ThT assays monitoring the formation of amyloid-like fibrils by teverelix under different conditions. (A) ThT assay of 0.2–3 mg/mL Tv in 25 mM citrate at pH 3.7 at 37 °C. (B) ThT assay of 0.1 mg/mL Tv in 25 mM citrate pH from 3.0 to 5.0 at 37 °C. The fluorescence data has been normalized, see Materials and Methods for further details. No half-life time (*t*_1/2_), lag time (*t*_lag_) and apparent growth rate (*k*) values were calculated at pH 3.0. For pH 4.0, these values were 748 min (*t*_1/2_), 308 min (*t*_lag_) and 0.0046 (*k*). For pH 5.0, these values were 652 min (*t*_1/2_), 64 min (*t*_lag_) and 0.0034 (*k*). (C) ThT assay of 0.5 mg/mL Tv in 100 mM citrate pH 3.0 with 0 to 11.0 mM TFA added at 37 °C. The fluorescence data has been normalized, see Materials and Methods for further details. (D) ThT assay (normalized) of 0.5 mg/mL Tv in 100 mM citrate pH 3.0 with 0–11 mM NaCl at 37 °C. (E) Normalized half-life (*t*_1/2_) versus [TFA] or [NaCl]. (F) Normalized apparent growth rate (*k*) versus [TFA] or [NaCl]. The kinetic parameters *t*_1/2_ and *k* were obtained from the best fit of the triplicate ThT assays shown in (C) and (D) to eqs 1 and 2. The error bars in parts (E) and (F) are the standard deviations based on triplicate data.

### Tv Concentration Influences the Kinetics of the Tv Fibril Formation

The ThT assays of a range of Tv concentrations from 0.2 to 3 mg/mL at pH 3.7, which were acquired in a 96-well plate in a fluorescence plate reader, are shown in Figure 4A. For the lowest concentration of Tv studied, 0.2 mg/mL (red), typical sigmoidal kinetics consistent with a nucleation–polymerization mechanism were observed. At pH 3.7, all higher peptide concentrations had a nonzero initial ThT intensity and no lag phase was observed, indicating that fibrils had already formed in the solution before the ThT plate reader assay was started. N.B. it took approximately 30 min to prepare the samples and load them onto the plate reader before the program was initiated and detection began. Thus, in these 30 min, for most Tv concentrations at pH 3.7, fibril formation is rapid, and the lag phase is over within the 30 min deadtime of the experiment and thus is not observed (Figure 4A). Notably, the initial and final ThT intensities are proportional to the Tv concentration, suggesting that ThT fluorescence intensity is a good measure of fibril formation and that the equilibrium lies well over toward that of the fibril (i.e., there is relatively little of the starting state (be it a monomer/dimer) left in solution at the end of the reaction). Additionally, from these data, it can be observed that the higher the Tv concentration, the shorter the time required to reach the final plateau, i.e., shorter *t*_1/2_. Though it is difficult to get the actual *t*_1/2_, *t*_lag_, and *k* values from fitting data at higher Tv concentrations due to the nonsigmoidal curves, it is clear from the data obtained that Tv concentration is an important factor in determining the rate of fibril formation as expected for a peptide that forms amyloid fibrils through a nucleation–polymerization mechanism.

In addition, to check that Tv fibrils formed following a nucleation–polymerization mechanism at higher peptide concentrations, additional experiments were undertaken monitoring ThT fluorescence in a single cuvette in a standard fluorimeter. This enabled us to have a shorter dead time, as only one sample needed to be prepared at one time. Two higher concentrations of Tv, 4 and 5 mg/mL, were used, and, in these cases, lag phase and sigmoidal kinetics were observed (Figure S6), consistent with a nucleation–polymerization mechanism. Unfortunately, as the agitation used in the plate reader assays could not be reproduced in the fluorimeter, the results could not be compared directly to those obtained above.

### pH Greatly Affects the Rate of Tv Fibril Formation

The kinetics of fibril formation were investigated at pH 3.0, 4.0, and 5.0 at a peptide concentration 0.1 mg/mL Tv using citrate buffer to maintain the pH throughout the reaction. The results are shown in Figure 4B. No ThT signals were observed at pH 3.0 at any peptide concentration, suggesting no, or very few, fibrils form at this low pH. In contrast, at pH 4.0 and 5.0, a sigmoidal increase in ThT fluorescence was observed and the data were fit to eqs 1 and 2 to obtain the kinetic parameters *t*_1/2_, *t*_lag_, and *k*, which are 12.5 ± 0.2 h, 5.1 ± 0.3 h, and 0.0046 ± 0.0001, respectively, at pH 4.0, and 10.9 ± 0.4 h, 1.1 ± 0.5 h, and 0.0034 ± 0.0002, respectively, at pH 5.0. These data establish that the rate of fibril formation is also pH-dependent and faster at higher pH values, consistent with the cac measurements described above. These results suggest that the pyridinium side chain in Tv must be deprotonated for the formation of critical species on the pathway to forming fibrils, e.g., an aggregate that can act as a nucleus, as well as in the fibril itself.

### Concentration of the TFA Counterions Influences the Kinetics of Tv Fibril Formation

The teverelix used was the TFA salt, and it is known that the molar ratio of TFA to Tv is approximately 2.1 or 2.2 to 1 in its lyophilized state.^1^ It is also known that only the TFA salt forms a microcrystalline state at high concentrations.^9^ Thus, counterion TFA may also play a role in the self-assembly of Tv. The effect of different TFA concentrations on the rate of fibril formation was investigated (Figure 4C). *t*_1/2_ values decrease and *k* increase with increasing TFA concentration, suggesting that TFA plays a role in fibril formation. However, as increasing the TFA concentration affects the ionic strength of the solution, a series of control experiments using NaCl were also undertaken to establish whether ionic strength also affected the rate of fibril formation (Figure 4D). These data showed a similar trend to TFA; however, the effects of NaCl were smaller than for TFA. The results for NaCl agree with other studies, which have shown that ionic strength frequently impacts the rate of fibril formation.^41–43^ To compare the effects of TFA and NaCl quantitatively, the kinetic parameters obtained from the best fit of the ThT assays shown in Figure 4C,D to eqs 1 and 2 were normalized, and the results for TFA and NaCl are shown together in Figure 4E,F. It is clear that TFA and NaCl both result in the same overall trends, but the effect of TFA is considerably larger than that of NaCl. These results suggest a specific role of TFA in stabilizing key species on the aggregation pathway such as oligomers, nucleus, and/or fibrils. The fact that the TFA concentration also affects the apparent growth rate indicates that Tv may add to an elongating fibril as the TFA salt.

### Characterization of Freshly Prepared Samples of Tv under Conditions Where Fibril Formation Is Slow

Having established the conditions under which Tv forms fibrils very slowly, i.e., low Tv concentrations and low pH (<4.0), experiments were undertaken under these conditions to characterize the starting state of the peptide in freshly prepared solutions before any significant self-assembly had occurred. These samples were then characterized using size-exclusion chromatography (SEC) and far- and near-UV CD.

### Oligomeric State of Tv in Freshly Prepared Solutions: SEC Experiments

Size-exclusion chromatography was used to determine the oligomeric species populated in freshly prepared solutions under conditions where self-assembly into fibrils is slow, i.e, low peptide concentrations (1.0 mg/mL) at pH 3.0 (Figure 5A). Two peaks were identified with elution volumes at 17.4 and 19.9 mL, and based on the calibration curve shown in Figure S7, the major peak eluting at 19.9 mL (fraction A) is from what is likely to be a dimer, while the minor peak eluting at 17.4 mL (fraction B) is from what is estimated to be a pentamer. N.B. note that as the calibration curve was calculated with globular proteins and Tv is a peptide with what is likely to be an extended (β) conformation, the oligomeric states are only approximate. The pentamer may well be either a tetramer or hexamer, given that the smallest stable oligomer observed is highly likely to be a dimer. As expected, as the Tv concentration increases from 0.5 to 1 mg/mL, the proportion of the dimer decreased, while the proportion of the pentamer increased (Figure 5A).

**Figure 5:**
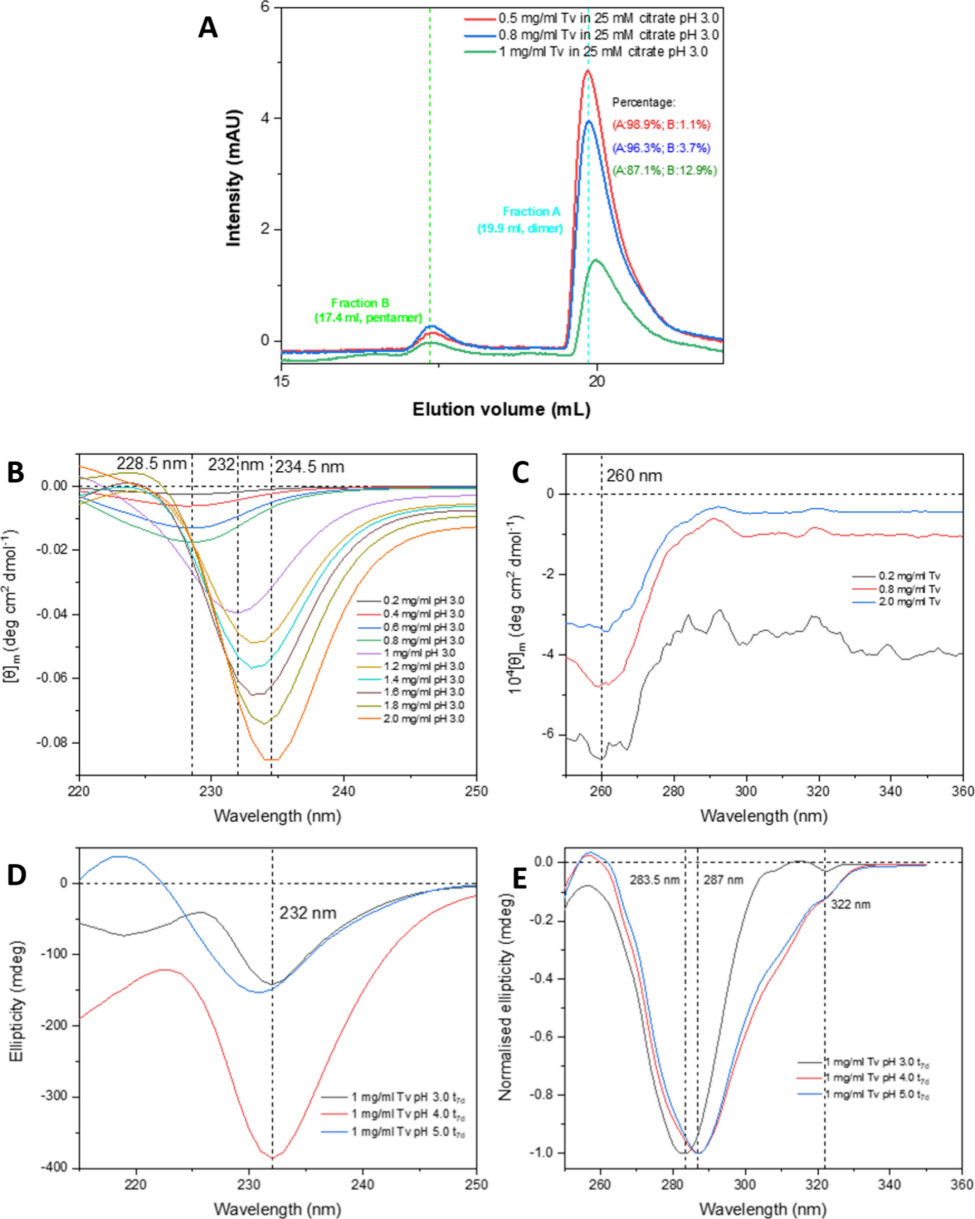
SEC profile of freshly prepared Tv and far/near-UV CD results. (A) Elution profile of Tv dimers and pentamers on a Superose 12 Increase 10/300 column in 25 mM citrate at pH 3.0. Elution profiles of freshly prepared 0.5, 0.8, and 1 mg/mL Tv in 25 mM citrate at pH 3.0 are overlaid. As the concentration of Tv increases, the intensity of dimers (19.9 mL) decreases, indicating a conversion from dimers to larger species. The intensity of the pentamer (17.4 mL) remains steady regardless of the Tv concentration. The percentage of each fraction is indicated in corresponding color. (B) Far-UV CD of freshly prepared 0.2, 0.4, 0.6, 0.8, 1.0, 1.2, 1.4, 1.6, 1.8, and 2 mg/mL Tv with 25 mM citrate at pH 3.0. All samples were prepared and measured within 5 min. A shift in the single minimum from 228.5 to 234.5 nm was observed as the concentration increased from 0.2 to 2 mg/mL. N.B. there was no peak shift from 0.2 to 0.8 mg/mL Tv. (C) Near-UV CD of freshly prepared 0.2, 0.8, and 2 mg/mL Tv in 25 mM citrate at pH 3.0. All samples were prepared and measured within 5 min. A single minimum at 260 nm was observed for all samples. (D) Far-UV CD of seven-day incubated 1 mg/mL Tv with 25 mM citrate at pH 3.0, 4.0, and 5.0. A single minimum at 232 nm (consistent with B) was observed for all samples. (E) Near-UV CD of seven-day incubated 1 mg/mL Tv with 25 mM citrate at pH 3.0, 4.0, and 5.0. Single minima at 283.5 nm for pH 3.0 and 287 nm for pH 4.0 and 5.0 were observed. In addition, a small shoulder at 322 nm was observed for all samples. The data were normalized based on the largest ellipticity value for each sample to make it easier to compare.

### Freshly Prepared Solutions of Tv at Low pH: Far- and Near-UV CD Experiments

In Figure 5B, the far-UV CD spectra of freshly prepared solutions of Tv at ten different peptide concentrations from 0.2 to 2 mg/mL at pH 3.0 are shown, and a single minimum shifting from 228.5 to 234.5 nm was observed. This is consistent with the naphthalene side chain in Tv being in a fixed conformation and chiral environment in the small oligomers (dimers/pentamers) that are formed in freshly prepared solutions at low pH. The shift in the minimum to higher wavelengths on increasing the peptide concentration indicates that the environment around the naphthalene changes as the equilibrium position of the system moves away from dimers toward larger oligomeric forms, consistent with the results from the SEC experiments.

The near-UV CD spectra for three different Tv concentrations at pH 3.0 were also recorded at three different peptide concentrations (Figure 5C), and a single minimum at 260 nm, which did not shift position over the concentration range 0.2–2 mg/mL, was observed. In this case, the near-UV CD spectrum comprises possible signals from the tyrosine, pyridine, phenylalanine, and naphthalene side chains. This result establishes that some of these aromatic side chains are already in a fixed, chiral environment in the freshly prepared sample (dimer/pentamer); however, it is not possible to say which.

Both far- and near-UV CD confirm that some of the aromatic side chains in Tv are fixed in a chiral environment in the starting state before any self-assembly has occurred, i.e, freshly prepared samples at pH 3.0. This is consistent with the SEC results, which show that the predominant species in solution under these conditions are dimers and other small oligomers (possibly pentamers).

### Characterization of the Amyloid Fibrils of Tv Using Far- and Near-UV CD and FT-IR Experiments

Far- and near-UV CD were also used to characterize the environment of the aromatic side chains of Tv in the fibrillar state. For this, three samples of 1 mg/mL Tv at pH 3.0, 4.0, and 5.0 were studied after a seven-day incubation at 25 °C under quiescent conditions. In the far-UV CD spectra, a single minimum at 232 nm was observed in all cases (Figure 5D), which is between wavelengths of 228.5 and 234 nm, which were observed for the dimer and pentamer. This result suggests that the environment of the naphthalene residue remains similarly buried in the fibril compared with the dimer/pentamer starting state but may have a slightly different environment, although it is not possible to say exactly what is different. In contrast, the near-UV CD spectra of Tv recorded after a seven-day incubation at pH 3.0, 4.0, and 5.0 (Figure 5E) were all different from the starting state observed at pH 3.0 (Figure 5C). N.B. near-UV CD spectra of the starting state at pH 4.0 and 5.0 could not be recorded as fibril formation is relatively fast at higher pH values. Instead of a minimum at 260 nm, a major minimum at 290 nm and a smaller minimum at 322 nm were observed (Figure 5E). These results indicate that at least one of the tyrosine, pyridine, or phenylalanine side chains undergoes a conformational change/change in the environment on fibril formation.

FT-IR was also used to check for any significant differences in the secondary structure in the lyophilized Tv powder (created directly after its initial synthesis) and lyophilized Tv fibrils. Two major peaks were identified for both samples. The amide I band was at 1643 cm^–1^ and the amide II band at 1535 cm^–1^ (Figure S8), which suggests that the peptide chain is in an extended conformation and adopts a β-structure in both samples.

### Mechanism of Amyloid-like Fibril Formation by Teverelix

The results described above can be used to propose a model for the formation and structure of amyloid-like fibrils by Tv. We believe that it is likely that the mechanism and structure of fibril formation we have characterized in vitro here are like the process that occurs in vivo after injection of Tv. From studies at low Tv concentrations and low pH, we know that the peptide exists in solution in a largely dimeric form, which we propose is stabilized by the burial of some of the large hydrophobic side chains including the naphthalene side chain and possibly some of the other hydrophobic side chains and aromatic groups (Figure 6A). This dimer is in equilibrium with a slightly larger oligomeric state, given the apparent instability of the monomer, likely to be either a tetramer or hexamer. In this state, the naphthalene side chain group has changed its environment somewhat, as seen by the observed shift in the wavelength minimum in the far-UV CD. Most likely, it has undergone further burial. As the population of the tetramer/hexamer increases with peptide concentration, as does the rate of fibril formation, we think it likely that the tetramer/hexamer is on the pathway to fibril formation; however, without further extensive experiments, it is impossible to rule out other more complex pathways, e.g., involving different on- and off-pathway oligomers.

**Figure 6:**
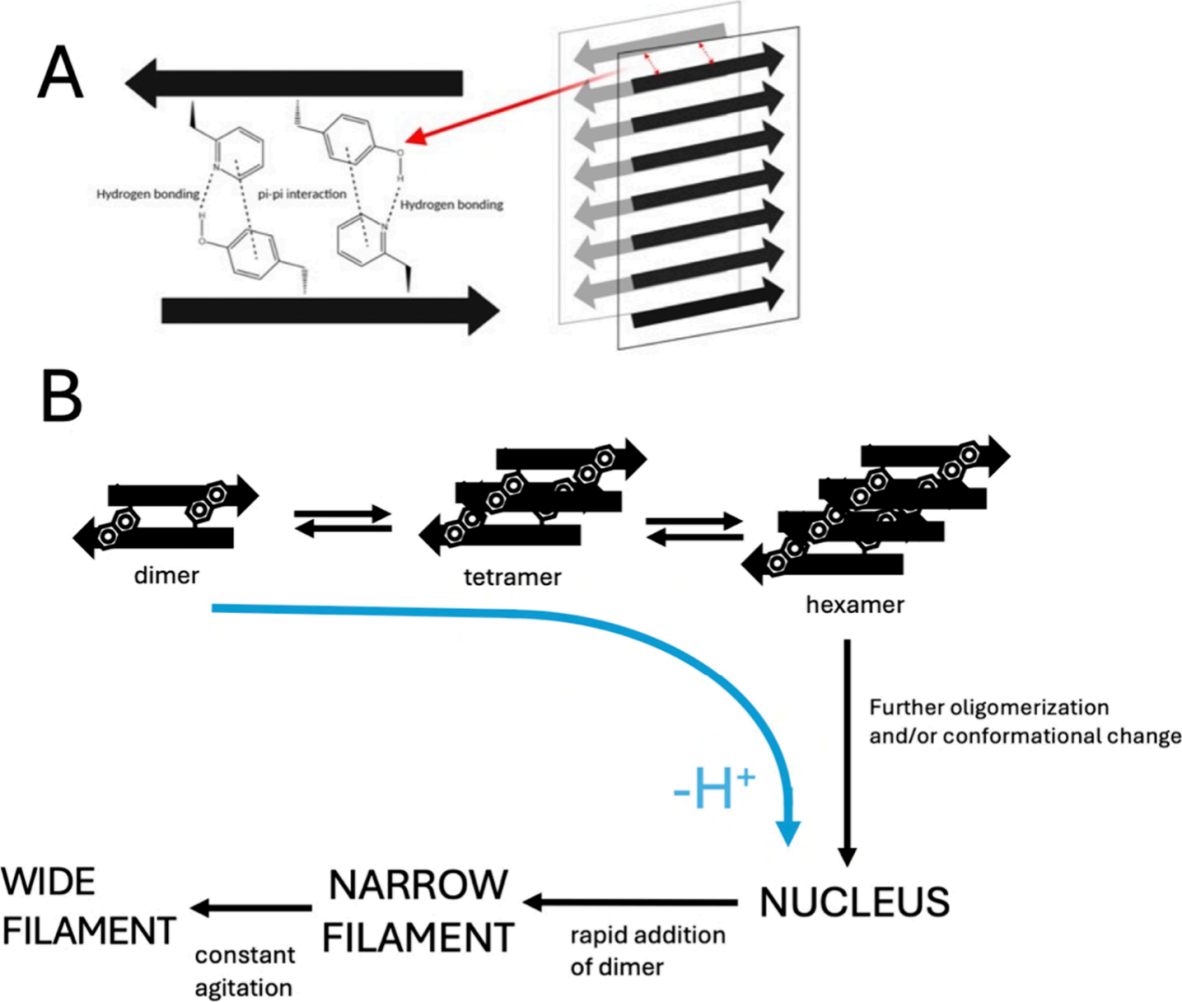
Schematic showing a putative structure of Tv within a narrow amyloid-like filament and a possible assembly pathway to the fibrillar state from the dimeric starting point. (A) Putative structure of a Tv dimer within a narrow amyloid-like filament. The double-arrow red dashed lines show possible hydrogen bonding and π–π interactions between each monomer within the dimeric unit within the narrow filaments. It is proposed that the deprotonated state of the pyridinium side chain hydrogen bonds with the tyrosine side chain, as inferred from the pH dependence of fibril formation observed. (B) Potential model of the self-assembly of dimeric teverelix into narrow and wide filaments (both amyloid-like fibrillar states capable of binding ThT). The dimeric state of Tv (in this state, the naphthalene side chain is already buried; however, the other aromatic side chains including the pyridinium and tyrosine are not fully buried). Dimers then form tetramers and, through association of another dimer, hexamers in which there has been some conformational change/burial of additional hydrophobic surface area. These then either form larger oligomers and/or undergo a conformational change to form a critical nucleus, which then rapidly elongates by addition of a dimer of Tv into either the narrow filaments observed by TEM or wider filaments consisting of two narrow filaments (also observed by TEM) if there is sufficient agitation. The nucleus likely elongates by the addition of a dimer of Tv. The blue curved arrow indicates that there is a deprotonation step during fibril formation, although it is not known exactly when this occurs.

Fibril formation occurs by the very well-established nucleation–polymerization as shown by the sigmoidal ThT kinetics obtained under some conditions as well as the fact that key kinetic parameters such as *t*_1/2_ and *t*_lag_ clearly depend upon Tv concentration. It is not possible to say exactly what size or structure the critical nucleus is. However, given that fibril formation is known to be rapid after formation of a nucleus and that there are measurable populations of dimers and tetramers/hexamers in the starting state it is possible that the nucleus is a larger oligomer or, alternatively, it could also be that the tetramer/hexamer has to undergo a relatively slow conformational change to form a state capable of rapid elongation and growth (Figure 6B). Again, given the relative instability of the monomer and stability of the dimer, it is likely that nuclei/fibrils elongate by addition of the dimer not the monomer (although we cannot rule out addition of the tetramer/hexamer). The results of the kinetic experiments using different concentrations of TFA indicate that the nucleus must contain TFA molecules and that TFA is also involved in the elongation step, which we assume occurs by the addition of dimers of Tv closely associated with TFA molecules.

Unusually for peptides that form amyloid-like fibrils, in the absence of significant agitation, the narrow filaments of Tv are stable and wide filaments consisting of two or more narrow filaments only form if agitation is constant. We propose that this may be directly due to the fact that a dimer, not a monomer, is the starting state. Thus, within a narrow filament that consists of dimers, there is significant burial of the hydrophobic surface area between the two extended β-sheets that have formed. Presumably, some further hydrophobic surface areas may be buried on formation of the wide filaments from the narrow filaments, but it appears that this may not be as extensive as the burial that occurs on narrow filament formation. Our results show that the Tv narrow filaments adopt a typical amyloid-like cross-β-structure as shown by X-ray fiber diffraction and FT-IR. Most importantly, the pH dependence of the cac and kinetics suggests that it is likely that the pyridinium side chain is deprotonated in the fibrillar state and may act as a H-bond acceptor with what we think maybe the tyrosine side chain (Figure 6A). However, we stress that this is a putative structure only.

### Comparison of the Self-Assembly of Teverelix with Other GnRH Antagonists Including Degarelix

Although relatively few studies on the self-assembly of teverelix prior to this one have been reported, a few papers on the self-assembly of other GnRH antagonists have been published. Early studies on both degarelix and LXT-101, another GnRH antagonist, observed the peptide to form a depot in vivo after injection^45,46^ similar to what is seen for teverelix. Further in vitro studies on degarelix established that residue 7 in the peptide (Leu in degarelix and teverelix) is critical in determining the nanostructures that the peptide can form.^15^ Both fibrils, which bound to Congo Red, suggesting they are amyloid in nature, as well as vesicles with dimensions in the order of tens of nanometers, were observed by TEM depending upon the residue at position 7.^15^ Interestingly, the fibrils formed by degarelix had widths reported to be in the range of a few nanometers and therefore somewhat smaller than those we observe here for teverelix. The two peptides differ from each other at positions 5 and 6, which are l-Tyr-D-Hcit (a carbamylated lysine side chain) in teverelix and 4Aph­(L-hydroorotyl)-D-4Aph­(Cbm) in degarelix. Thus, degarelix has a considerably larger side chain than teverelix at position 5, while the only difference at position 6 is the substitution of a −CH_2_–CH_2_– group in the lysine side chain in teverelix with a benzyl group in degarelix, which has the potential to alter the side chain in terms of sterics and hydrophobicity. Either/both of these substitutions could cause the differences in self-assembled structures observed, both in terms of the width of fibrils and possibly the formation of vesicles (although this is most likely due to changes in the side chain of residue 7). Other studies with degarelix have focused on the interaction of the peptide with polyanions, including alginate and carboxymethyl cellulose.^16,47^ Degarelix in the absence of the polyanion was shown to form twisted fibrils, again potentially amyloid-like in nature. Upon addition of the polyanion, these types of aggregates were observed to dissolve and a stable polyanion-degarelix complex formed, which varied somewhat in size, shape, and stability dependent upon the polyanion used.^16,47^ A direct comparison with teverelix cannot be undertaken, as we did not perform any experiments with polyanions in this study.

Self-assembly studies on LXT-101 using different solution conditions also established that in water the peptide forms stable fibrils, while in the presence of excipients such as mannitol, dextrose, or NaCl, less stable vesicles were observed by TEM. These results contrast what we find for teverelix here, where the addition of mannitol or NaCl has no effect on the nanostructures formed, and amyloid-like fibrils are always observed. Thus, we can speculate that teverelix has a higher propensity for amyloid fibril formation than LXT-101 and possibly degarelix.^48^

## Conclusions

In this study, the amyloid-like identity of Tv fibrils was first confirmed using X-ray fiber diffraction, and the morphology of the fibrils was studied by TEM. In contrast to many other amyloid-forming peptides, narrow filaments were the major species populated over a wide range of conditions for Tv, with wider fibrils only being formed in any significant amount when agitation was continuous. From the studies of far/near-UV CD and intrinsic fluorescence, the naphthalene side chain of Tv is buried and in a fixed conformation even in the starting state, which was shown by SEC to be largely dimeric (with a small proportion of tetramers/hexamers; the population of which increased with Tv concentration). A small change in the environment around the naphthalene side chain was shown to occur on forming the slightly larger soluble oligomers compared with the dimer, while a large change in the environment of the pyridinium and other aromatic side chains, including the tyrosine side chain, occurs on fibril formation. Interestingly, there appears to be relatively little conformational change in the secondary structure of Tv between the lyophilized powder and the fibrils, both states containing mainly β-structure as shown by FT-IR. This suggests the starting conformation of Tv is already in a highly aggregation-prone state and maybe one reason why Tv forms fibrils so rapidly under certain conditions.

The kinetics of fibril formation by Tv were studied in detail to understand more about the factors that affect this process. Under conditions in which the fibrils formed sufficiently slowly, sigmoidal kinetics were observed consistent with a nucleation–polymerization mechanism common to many amyloid-forming peptides. The kinetics were peptide-concentration-dependent, with faster aggregation occurring at higher Tv concentrations. The stability (as shown by the critical aggregation concentration*)* and the kinetics of fibril formation were both shown to depend critically on pH. The critical aggregation concentration decreases with increasing pH, and the rate of aggregation increases significantly at higher pH values such that by pH 5.0 the self-assembly of Tv is so fast it can only be monitored at low Tv concentrations. Additionally, it was found that the counterion TFA plays an important role in fibril formation, with results suggesting that not only it is needed to stabilize key species on the self-assembly pathway and that it is also stably incorporated into the fibrillar state. To the best of our knowledge, this is the first paper to study in detail the formation of fibrils of this therapeutic peptide, a process which is critical to the long-acting action of this drug in vivo.

## Supplementary Material



## Notes

TEM images of fibrillar states formed over a wide range of conditions, pH changes on fibril formation, calculation of number of protons released on fibril formation, estimation of critical aggregation concentrations using ThT, ThT kinetics following fibril formation at 4 and 5 mg/mL Tv (PDF)
