# Supplementary material for: Mechanism of Self-Assembly of the Gonadropin Releasing Hormone Antagonist Teverelix into Amyloid Fibrils
Source: Mol Pharm. 2025 Dec 18;23(1):164–76. doi: 10.1021/acs.molpharmaceut.5c00578 (PMC12776573; doi:10.1021/acs.molpharmaceut.5c00578)
Supplement: Supplementary file 1 [file mp5c00578_si_001.pdf]

Mechanism of Self-assembly of the Gonadropin Releasing Hormone Antagonist Teverelix into Amyloid Fibrils

Xinyang Li1, Louise C. Serpell2, Jens T. Bukrinski3, Francois Boutignon4, Carol M. MacLean5 & Sophie E. Jackson1*

[1]Yusuf Hamied Department of Chemistry, Lensfield Road, Cambridge, CB2 1EW, UK

[2]Sussex Neuroscience, School of Life Sciences, University of Sussex, Brighton, BN1 9QG, UK.

[3] CMCAssist ApS, Ole Maaløes Vej 3, Copenhagen N 2200, Denmark.

[4] Boutignon & Partners, 2 rue Michel Renaud, Biopole Clermont-Limagne, 63360 Saint Beauzire, France.

[5]Antev Ltd, Ibex House, Baker Street, Weybridge, Surrey KT13 8AH, UK.

***Corresponding author. E-mail: sej13@cam.ac.uk**

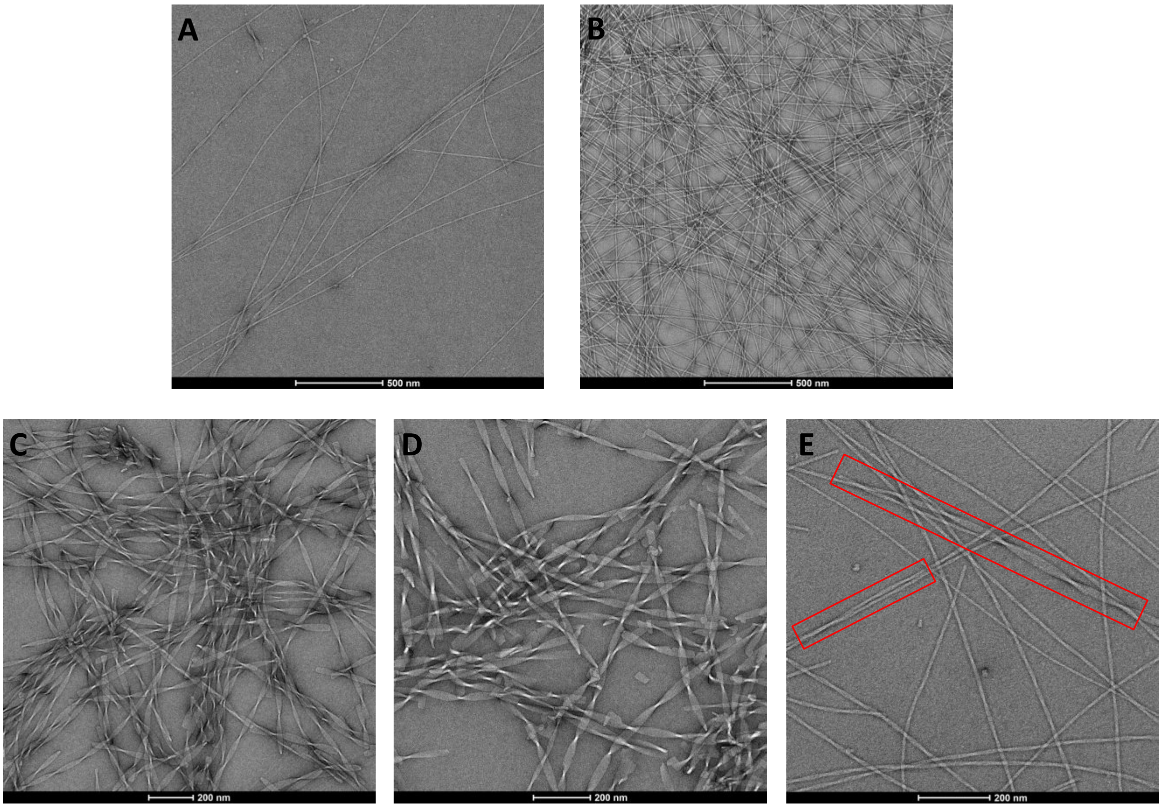

Figure S1. Summary of Tv observed under TEM. (A&B): TEM images of freshly prepared 0.2 mg/mL (pH 4.1) and 1 mg/mL (pH 3.7) Tv in ddH_2O at room temperature. Tv samples were loaded on the grid and imaged within 30 mins. Fibrils were observed in both samples and the fibril density is proportional to the Tv concentration. **(C&D):** 1 mg/mL Tv incubated in 25 mM citrate pH 4.0 for one and seven days at 37 °C with agitation. In **C**, a mass of Tv wide filaments (about 20 nm width) were observed, which had the same morphology as those observed in **Figure 1B**. The sample was further incubated in **D**. The Tv wide filaments were stable after seven-day incubation, though few dissociated into fragments. **(E):** TEM image of 0.9 mg/mL Tv (about pH 3.7) in ddH_2O incubated for five hours at room temperature without agitation. Two wide filaments assembled into one higher order structure in high resolution were captured, which is highlighted in the red rectangles.

Tv in 25 mM pH 3.0 citrate buffer	Tv Conc. (mg/ml)	pH of freshly prepared sample	pH of sample incubated for 7 days
	0.2	3.04	3.03
	0.5	3.03	3.03
	1	3.02	3.03
	2	3.01	2.96
	3	2.98	2.92
	4	2.99	2.91
	5	3.00	2.88

Table S1. pH values of freshly prepared and seven-day incubated Tv samples from 0.2 to 5 mg/mL in 25 mM citrate pH 3.0 at room temperature. Though pH drop was observed after seven-day incubation, the pH-control was acceptable for all samples by using the 25 mM citrate pH 3.0 buffer.

Calculations based on the pH decrease shown in Figure 3A

Taking the 2 mg/mL Tv sample as an example, the pH decreases from 3.48 to 2.87 during the seven-day incubation. The protons required to cause the pH drop = $10^{-2.87} - 10^{-3.48}$ = 0.00135 – 0.00033 = 1 x 10^{-3} M. Therefore, for 1 L sample, 1 x 10^{-3} mol protons are required to result in the decrease in pH observed.

Considering the presence of TFA, the mole amount of pyridine/pyridinium side chains present in a 2 mg/mL sample are = 1.7 mg/mL/1459 g/mol = 1.2 x 10^{-3} mol. Assuming all the pyridyl side chains exist in their protonated form as pyridinium ions and they are all deprotonated on fibril formation then, for a 1 L sample, the maximum proton release would be 1.2 x 10^{-3} mol, which is close to that observed for the decrease in pH observed (1 x 10^{-3} mol).

Estimation of the critical aggregation concentration (*cac*) of Tv

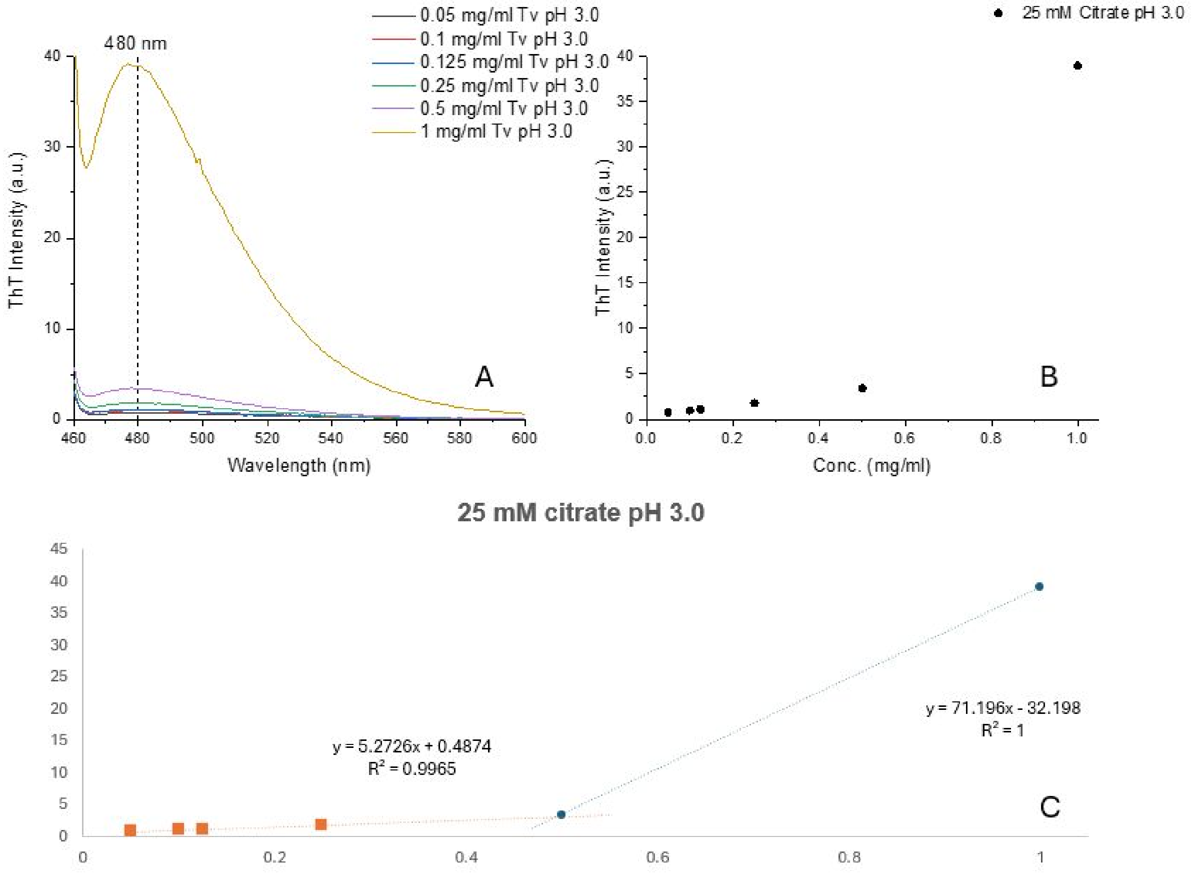

Figure S2. ThT fluorescence of different concentrations of Tv in 25 mM citrate pH 3.0 and estimation of the critical aggregation concentration. (A): ThT fluorescence spectra of ThT in the presence of different concentrations of Tv. **(B)**: ThT fluorescence intensity at 480 nm at different concentrations of Tv. **(C)**: Estimation of the critical aggregation concentration from the intersection of straight lines fitted to the data at low and high Tv concentrations. *Cac.* is approx. 0.5 mg/mL.

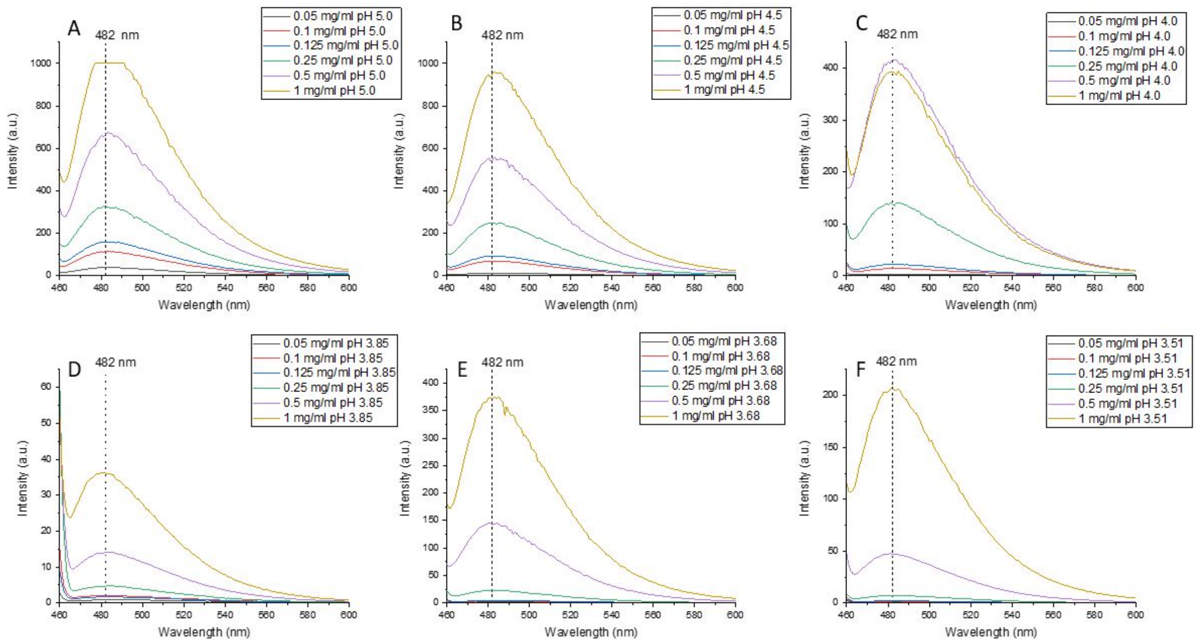

Figure S3. ThT fluorescence of freshly prepared 0.05 to 1 mg/mL Tv samples in 25 mM citrate from pH 3.51 to 5.0 at 25 °C (A to F). (A): pH 5.0, (B): pH 4.5, (C): pH 4.0, (D): pH 3.85, (E): pH 3.68 and (F): pH 3.51. Each measurement was done within 30 mins of dissolving the Tv powder in buffer.

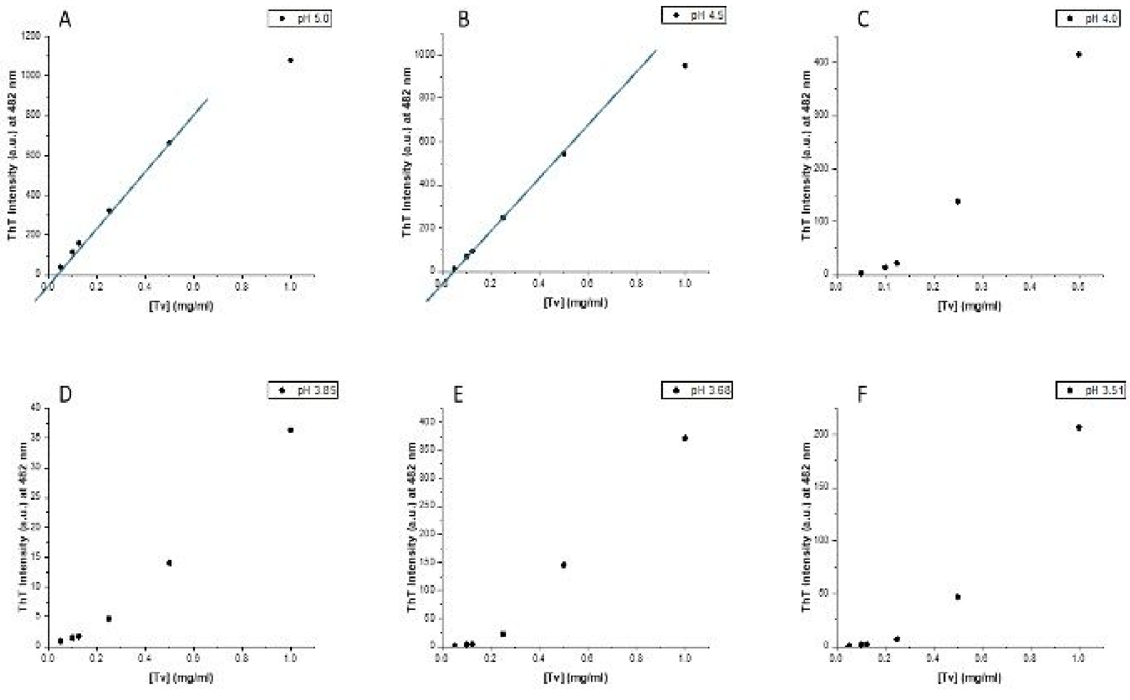

Figure S4. Analysis of ThT fluorescence data shown in Figure S3 (A to F) to estimate the critical aggregation concentration of Tv at different pH values. (A): pH 5.0, (B): pH 4.5, (C): pH 4.0, (D): pH 3.85, (E): pH 3.68 and (F): pH 3.51. Tv samples used were freshly prepared from 0.05 to 1 mg/ml in 25 mM citrate from pH 3.51 to 5.0 at 25 °C. The blue line shown in panels A & B is used to estimate the maximum *cac*.

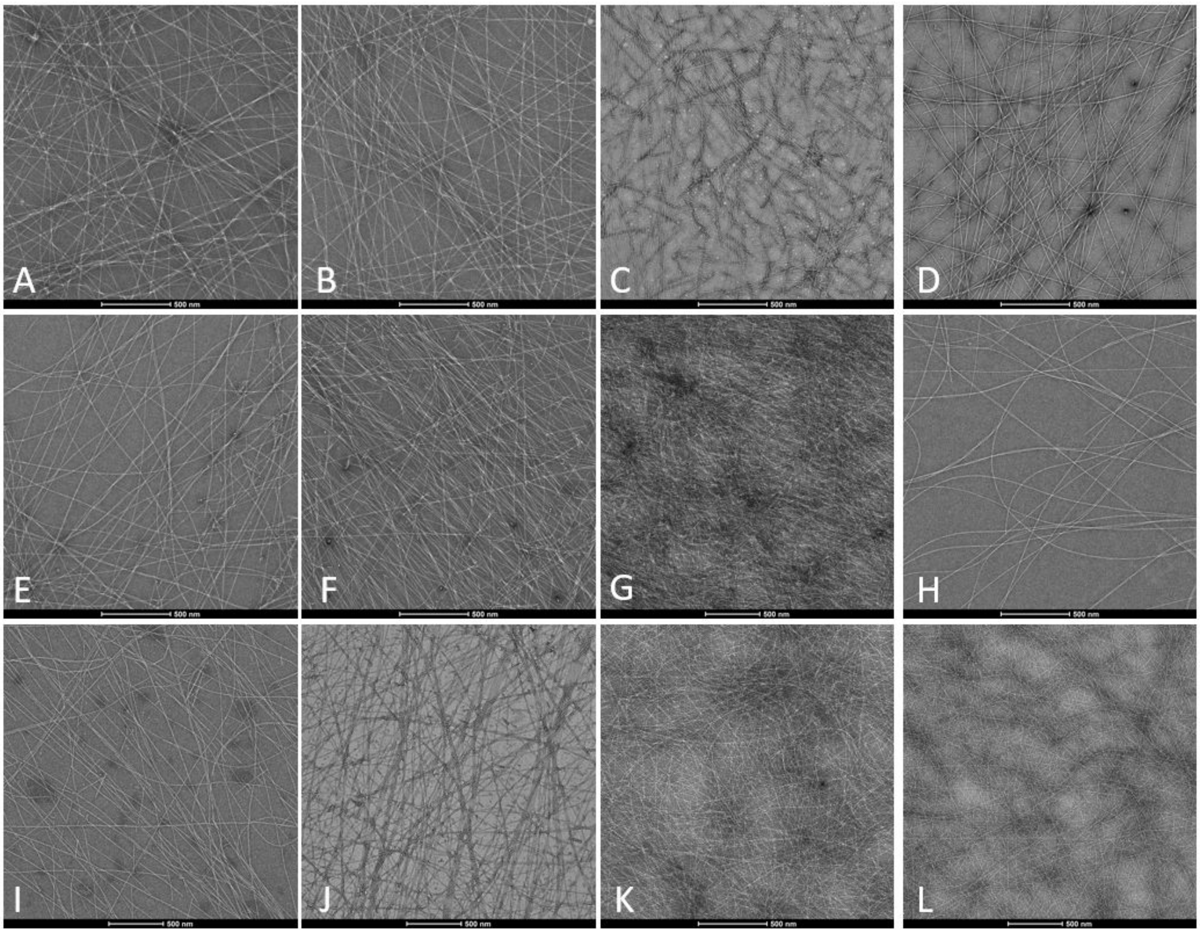

Figure S5. TEM images of fibril samples obtained at the end of ThT assays (after seven days) under various conditions. A: 0.2 mg/mL Tv in ddH$_2$O (pH 4.1). **B:** 0.5 mg/mL Tv in ddH$_2$O (pH 3.71). **C:** 1 mg/mL Tv in ddH$_2$O (pH 3.23). **D:** 2 mg/mL Tv in ddH$_2$O (pH 2.87). **E:** 3 mg/mL Tv in ddH$_2$O (pH 2.73). **F:** 4 mg/mL Tv in ddH$_2$O (pH < 2.73). **G:** 5 mg/mL Tv in ddH$_2$O (pH < 2.73). **H:** 10-fold dilution of sample used in **F** (3.71 < pH < 4.10). **I:** 10-fold dilution of sample used in **G** (pH 3.71). **J:** 2 mg/mL Tv in 25 mM citrate pH 3.0 with 5% *w/v* mannitol. **K:** 5 mg/mL Tv in 25 mM citrate pH 3.0. **L:** 5 mg/mL Tv in 25 mM citrate pH 3.0 with 5% *w/v* mannitol. All samples were obtained at the end of ThT assays after seven days of incubation with periodic agitation at 37°C.

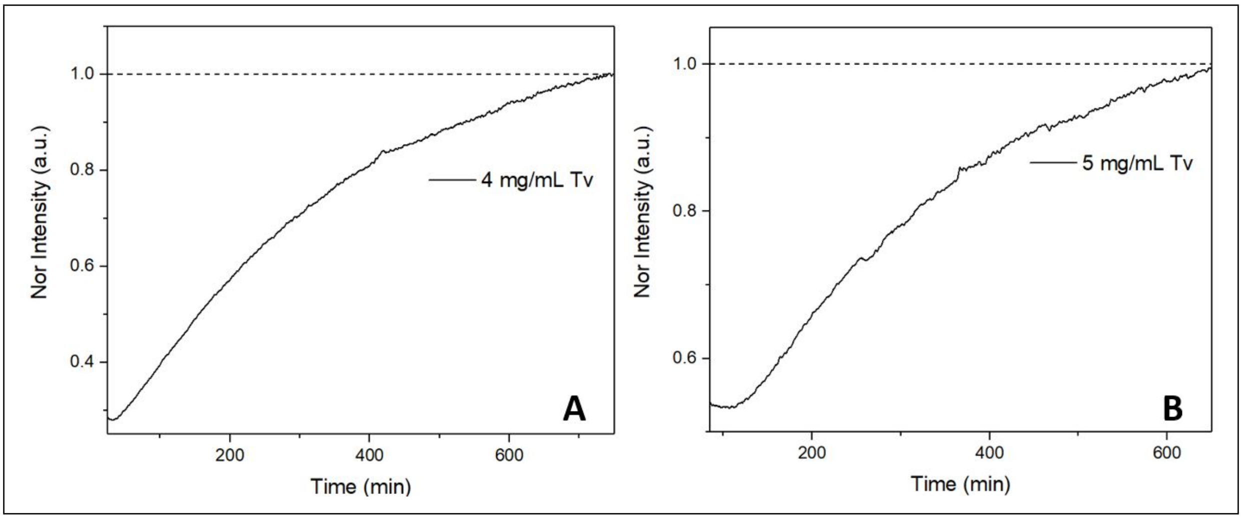

Figure S6. Sigmoidal kinetics of the aggregation of samples of 4 and 5 mg/mL Tv monitored by ThT fluorescence in a single-cuvette fluorimeter. The ThT fluorescence of 4 and 5 mg/mL Tv in 25 mM citrate at pH 3.7 at 37 °C. The deadtime was kept short (approx. two minutes). The final ThT concentration in the sample is 50 mM which is the same as used for ThT assays. Both curves were fitted to Equations 1 and 2. The fitted $t_{1/2}$ and k values for the data shown in **A** (4 mg/mL Tv) were 486 mins and 0.0029, respectively, whilst the values for the data shown in **B** (5 mg/mL) were 412 minutes and 0.0034, respectively. These values were not compared to the plate-reader ThT assay data since an periodic agitation was used in the plate reader, which was not possible for these ThT fluorescence experiments conducted in a standard fluorimeter.

Calibration of Superose 12 10/300 in 25 mM sodium phosphate buffer at pH 8.

Protein standard	M_W [Da]	Elution volume [mL]
Aldolase	158000	11.4
Conalbumin	75000	12.2
Ovalbumin	44000	12.6
Carbonic anhydrase	29000	13.9
Trypsinogen	24000	14.5
Ribonuclease A	13700	15.1
Chymotrypsin inhibitor 2	9265	15.9
Teverelix pentamer	7295	17.4
Teverelix dimer	2918	19.9

Table S2. Protein calibration standards and their elution volumes on a Superose 12 10/300 in 25 mM sodium phosphate buffer at pH 8.

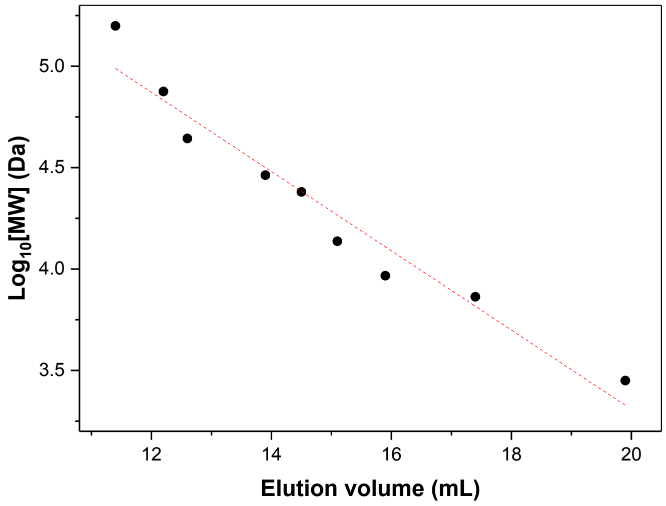

Figure S7. Calibration curve for the Superose 12 Increase 10/300 column on molecular weight. Calibrations on the molecular weight were performed in 25 mM sodium phosphate at pH 8.0 using a set of protein standards **(Table S2)** at room temperature. N.B. Tv samples (at different concentrations) have varying pHs due to the presence of TFA counterions, and the pH of citrate buffered Tv samples was 3.0. However, the calibration could not be done under acidic conditions. It is assumed that the calibration at pH 8 shown here applies at low pH values too. The equation of the linear regression fit (red dotted line) is shown below.

$$[\mathrm{Log}_{10}(M_W)] = -0.1943[V_e] + 7.2160$$

$$R^2 = 0.9513$$

In **Figure 5A** in the main text, two major peaks were identified in the elution profile of 0.8 mg/mL Tv in 25 mM citrate pH 3.0 at 17.4 mL (fraction B, 3%) and 19.9 mL (fraction A, 97%) respectively, using the equation above, which have molecular weights of approx. 6918 and 2240 Da. The former (17.4 mL, fraction B, 3%) likely corresponds to a Tv pentamer (7295 Da), while the latter to a Tv dimer (2918 Da).

Fourier transform infrared spectroscopy (FT-IR)

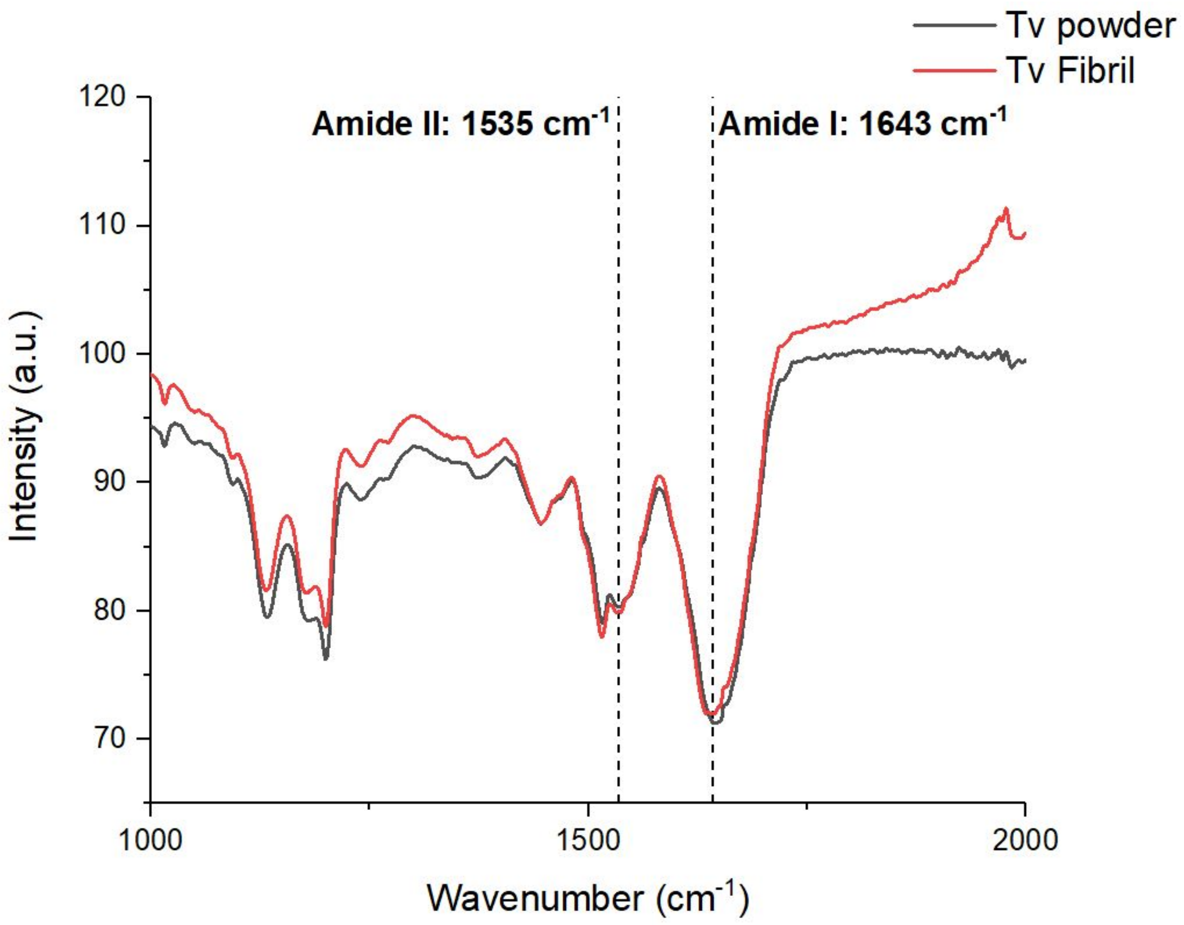

Figure S8. Overlay of the FT-IR spectra of Tv powder and lyophilised Tv fibrils. The Tv powder was stored in a -20 °C freezer and directly measured within 15 mins. The fibril powder was made from 1 mg/mL Tv in ddH_2O incubated for seven days at room temperature followed by overnight lyophilisation.
